# Andrographolide Mitigates Inflammation and Reverses UVB-Induced Metabolic Reprogramming in HaCaT Cells

**DOI:** 10.3390/ijms26136508

**Published:** 2025-07-06

**Authors:** Carolina Manosalva, Pablo Alarcón, Lucas Grassau, Carmen Cortés, Juan L. Hancke, Rafael A. Burgos

**Affiliations:** 1 Institute of Pharmacy, Faculty of Sciences, Universidad Austral de Chile, Valdivia 5090000, Chile; carolinamanosalva@uach.cl (C.M.); lucas.grassau@alumnos.uach.cl (L.G.); carmen.cortes@alumnos.uach.cl (C.C.); 2 Laboratory of Inflammation Pharmacology and Immunometabolism, Institute of Pharmacology and Morphophysiology, Faculty of Veterinary Sciences, Universidad Austral de Chile, Valdivia 5090000, Chile; pablo.alarcon@uach.cl (P.A.); jhanckeo@gmail.com (J.L.H.)

**Keywords:** andrographolide, photoaging, wound healing, keratinocyte, metabolism

## Abstract

Andrographolide (AP), a bioactive compound from *Andrographis paniculata*, is known for its anti-inflammatory and antioxidant properties, both essential for wound healing. However, its effects on energy metabolism during tissue repair and its role in UVB-induced photoaging remain poorly understood. This study explored AP’s multitarget therapeutic effects on wound healing under photoaging conditions (PhA/WH) using network pharmacology and experimental validation. Scratch wound assays showed that AP promoted keratinocyte migration in UVB-exposed HaCaT cells. Bioinformatic analysis identified 10 key targets in PhA/WH, including TNF-α, IL-1β, JUN, PPARγ, MAPK3, TP53, TGFB1, HIF-1α, PTGS2, and CTNNB1. AP suppressed UVB-induced pro-inflammatory gene expression (*IL-1β*, *IL-6*, *IL-8*, and *COX-2*) and inhibited the phosphorylation of ERK1/2 and P38, while enhancing Hypoxia-Inducible Factor-1alpha (*HIF-1α)* and peroxisome proliferator-activated receptors *(PPARγ)* expression. GC/MS-based metabolomics revealed that AP reversed UVB-induced disruptions in fatty acid metabolism, glycolysis/gluconeogenesis, and tricarboxylic acid (TCA) cycle, indicating its role in restoring the metabolic balance necessary for tissue regeneration. In conclusion, andrographolide modulates key inflammatory and metabolic pathways involved in wound repair and photoaging. These mechanistic insights contribute to a better understanding of the molecular processes underlying skin regeneration under photodamage and may inform future therapeutic strategies.

## 1. Introduction

Skin wound repair is a complex and metabolically demanding process that involves multiple cell and tissue lineages to meet homeostatic and regenerative requirements. Various factors, such as pressure, vascular insufficiency, chronic diseases, and aging, play an essential role in modulating wound metabolism by limiting fuel and oxygen availability. Failure to meet bioenergetic demands during wound healing contributes to the formation of chronic wounds [1].

Recent studies have demonstrated that UVB radiation impacts keratinocyte migration and hinders wound healing [2]. Previous reports have detailed the metabolic changes through which UVB rays inflict skin damage [1]. Exposure to UVB leads to a reduction in glycolysis, the tricarboxylic acid cycle, and fatty acid β-oxidation, while simultaneously increasing mitochondrial oxidative phosphorylation [3,4]. Similarly to UVB exposure, the chronic wound environment is hypoxic and is linked to a metabolic shift towards the less energy-efficient anaerobic metabolism [1]. Currently, research is underway to explore drugs capable of regulating metabolism in various pathological processes and offering therapeutic benefits for certain diseases [5].

The interaction between inflammation, metabolic reprogramming, and photoaging, along with several significant knowledge gaps, continues to be the subject of active investigation. Although it has been established that UVB exposure induces DNA damage and NF-κB-mediated inflammation, the mechanisms connecting inflammation to metabolic alterations in skin cells have not yet been fully elucidated [6,7,8]. A critical gap in our understanding is the role of inflammation in accelerating age-related skin deterioration and photoaging. Pro-inflammatory cytokines, such as IL-1β and TNF-α, not only disrupt metabolic pathways, but also compromise skin integrity and repair mechanisms. However, the specific metabolic changes driven by persistent inflammation in aging skin remain insufficiently explored [6,7,8].

Acute UVB exposure induces rapid activation of the MAPK signaling pathway, leading to the production of pro-inflammatory cytokines and COX-2 in keratinocytes. Evidence suggests that the activation of p38 and ERK1/2 mitogen-activated protein kinase (MAPK) is involved [9]. In addition, MAPK inhibitors have been shown to reduce skin inflammation [9]. The MAPK pathway can induce downstream activation of pro-inflammatory transcription factors, such as NF-κB, activator protein 1 (AP-1) and HIF-1α [10,11]. NF-κB and HIF-1α are closely related to the control of cellular metabolic reprogramming during inflammation [10,11]. These findings suggest that the use of MAPK or transcription factor inhibitors may potentially restore the altered metabolism in keratinocytes exposed to UVB radiation, thereby promoting wound healing. However, there is no evidence that drug modulators of metabolic reprogramming promote wound healing.

Andrographolide (AP) is a natural diterpenoid and the main active ingredient in the leaves and stems of *Andrographis paniculata*, a herbal medicine cultivated mainly in Asia [5]. It has become the subject of multiple studies owing to its antitumor, antiangiogenic, antioxidant, and anti-inflammatory properties. Recent studies have revealed that AP protects against photodamage induced by UVB radiation in the mouse skin by impeding inflammation, oxidative stress, and apoptosis [12]. AP offers significant advantages over non-steroidal anti-inflammatory drugs (NSAIDs) [5,12,13]. Although NSAIDs act primarily by inhibiting cyclooxygenase (COX) enzymes, AP modulates multiple signaling pathways, including NF-κB and MAPKs, leading to reduced cytokine production and oxidative stress without the adverse effects commonly associated with NSAIDs [14]. In addition, it enhances the activity of antioxidant enzymes and regulates key cell cycle proteins, such as p21 and p53, thereby influencing cell proliferation and apoptosis. These multifaceted actions make AP a promising alternative for the treatment of UVB-induced skin inflammation and oxidative damage [14,15,16].

For instance, a comprehensive review by Li et al. summarizes its anti-inflammatory, antioxidant, anticancer, antimicrobial, and antihyperglycemic properties, along with insights into its pharmacokinetics and safety [13]. A systematic review emphasizes its antimicrobial activity across bacterial, viral, fungal, and protozoal pathogens [17]. Additionally, recent reviews have explored its cardiovascular protective effects and anticancer mechanisms involving NF-κB, MAPK, PI3K/AKT/mTOR, and JAK/STAT pathways [18,19], many of which overlap with UVB-induced cellular responses. These findings underscore the relevance of further investigating andrographolide’s protective role in UVB-induced skin damage and metabolic reprogramming, as explored in our study.

In addition, andrographolide sodium bisulfate (ASB), a soluble derivative of andrographolide and sodium bisulfate, inhibits UV-ROS-induced inflammation and oxidative stress following skin cell damage by downregulating the NF-κB pathway and activating the Nuclear factor erythroid 2-like 2 (NRF2) pathway [20]. Moreover, AP has been proposed to modulate cellular metabolism, thereby contributing to its anti-inflammatory effects [5]. AP acts as a multitarget drug that inhibits the MAPK and NFκB/HIF-1α pathways, which could reduce metabolic reprogramming during inflammation. Recently, we demonstrated that AP reduced the expression of IL-6 and COX-2 and interfered with the NF-κB/HIF-1α pathway and expression of Lactate Dehydrogenase -A (LDHA) in synoviocytes [21].

In this study, we demonstrated that AP enhanced the migration of keratinocytes exposed to UVB radiation. Using network pharmacology analysis, we identified that AP targets proteins involved in the inflammatory process, such as IL-1β and COX-2, as well as transcription factors such as PPARγ and HIF-1α, which regulate cellular metabolism. These findings were validated through in vitro experiments, where AP was shown to reduce the expression of pro-inflammatory genes (*IL-1β*, *IL-6*, *IL-8*, and *COX-2*) induced by UVB while simultaneously increasing the levels of *PPARγ* and *HIF-1α*. In addition, we demonstrated that AP restored the energy metabolism disrupted by UVB exposure. This study provides an initial experimental approach to understanding the mechanisms by which AP may influence wound healing under photoaging conditions. In UVB-irradiated keratinocytes, AP modulates inflammatory responses and metabolic pathways associated with tissue repair. Although the results are promising, further research is needed to confirm its therapeutic relevance and fully elucidate its role in skin regeneration.

## 2. Results

### 2.1. Andrographolide Restored Migration of UVB-Irradiated HaCaT Cells

Directional cell movement contributes significantly to skin wound repair. It has recently been shown that UVB radiation inhibits keratinocyte migration, thereby altering wound healing [2]. To clarify whether AP can restore migration impaired by UVB irradiation, we treated UVB-irradiated HaCaT cells with 5.0 and 10 µM AP for 24 h using the scratch assay method. To support the selection of these concentrations, we performed additional viability assays using the CCK-8 method, which confirmed that both 5 µM and 10 µM AP were non-cytotoxic in our experimental conditions. In contrast, we observed a significant decrease in cell viability above 20 µM (Supplementary Figure S1), and therefore this concentration was not included in the migration experiments to avoid confounding effects due to cytotoxicity.

**Figure 1:**
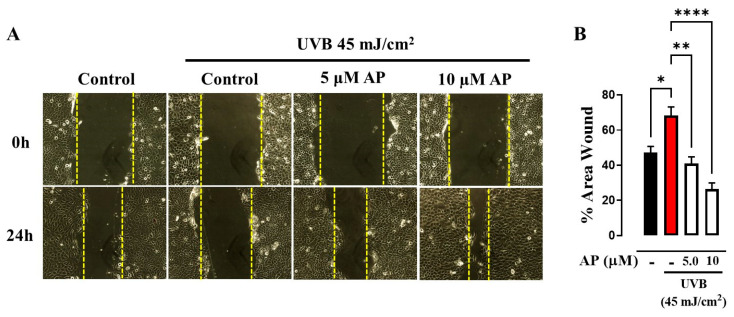
A scratch wound assay was performed on UVB-irradiated HaCaT cells, with or without AP treatment. (**A**) Representative images taken at 0 and 24 h post-wound induction for the control, UVB, and UVB + AP treatment groups. Yellow dashed lines denote the wound edges at each time point. (**B**) Quantification of the wound closure percentage at 24 h in the experimental groups. Data are presented as mean ± SEM; * *p* < 0.05, ** *p* < 0.01, and **** *p* < 0.0001 compared to the control group. *n* = 4.

The results demonstrated that non-irradiated cells had a significantly lower percentage of cell-free area than did UVB-irradiated cells. The percentages of these areas were 47.1% ± 3.058 and 68.2% ± 4.876, respectively. In contrast, we observed that treatment with AP significantly reduced the wound area compared with untreated irradiated cells. Treatment with 5.0 µM and 10 µM AP reduced the wound area to 40.9% ± 3.795 and 26.4% ± 3.516, respectively (Figure 1A,B). These results show that AP restores UVB-impaired keratinocyte migration, suggesting that AP plays a role in the treatment of UVB-induced photodamage.

### 2.2. Identification of Pharmacological Targets for Andrographolide in the Treatment of PhA/WH

**Figure 2:**
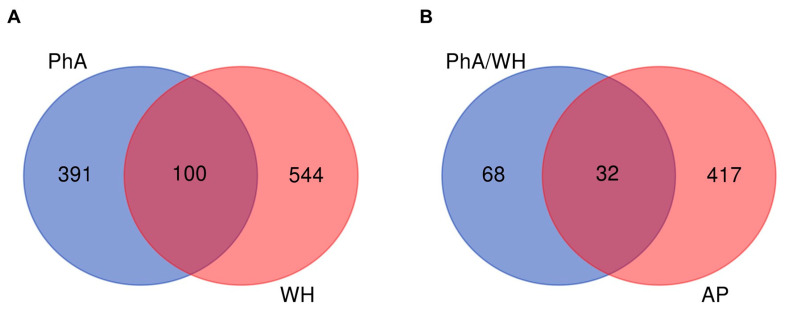
Venn diagrams depict the overlap of differentially expressed genes under different experimental conditions. (**A**) Comparison of genes modulated under photoaging (PhA) and wound healing (WH) conditions, highlighting the shared subset (*n* = 100) and genes unique to each condition. (**B**) Comparison of genes modulated during wound healing under photoaging (PhA/WH) conditions with andrographolide (AP) target genes, indicating a shared subset (*n* = 32) of genes unique to each condition. These analyses showed the degree of overlap and specificity of the gene expression profiles across the different experimental groups.

After removing duplicates, a list of 491 targets related to photoaging was generated using GeneCards and OMIM databases. For the wound healing process, 644 targets were identified by analyzing data obtained from OMIM, Disease GDa, CTD, and GeneCards. By intersecting both lists, 100 common targets were identified, highlighting the existence of a significant set of biomarkers and potential pharmacological targets shared between photoaging and wound healing (Figure 2A).

These findings underline the possibility of developing integrated therapeutic strategies that can simultaneously address these two conditions, considering that they share key biological processes such as inflammation, oxidative stress, and cell regeneration. Shared targets may play a fundamental role in identifying molecular mechanisms involved in the design of new treatments.

In contrast, 449 AP targets were predicted using Charité, SwissTarget, PharmMapper, TargetNet, and CTD platforms. By crossing the drug targets associated with photoaging/wound healing (PhA/WH) with those of AP, 32 common targets were identified (Figure 2B). To prioritize the 32 shared targets between the compound and disease, we also performed a protein–protein interaction network analysis using STRING and calculated centrality metrics in Cytoscape. Supplementary data present the 32 molecular targets identified as commonly modulated by andrographolide under UVB-induced cellular stress. For each target, the official gene symbol, full protein name, UniProt identifier, and a brief functional annotation are included. These annotations were retrieved using the UniProt batch retrieval tool (https://www.uniprot.org, accessed on 5 November 2024), based on manually reviewed entries from the Swiss-Prot database for Homo sapiens.

These results suggest that AP has the potential to influence the shared molecular pathways of photoaging and wound healing, thus reinforcing its value as a promising therapeutic candidate. Identification of these 32 shared targets may represent potential molecular targets for future studies aimed at developing combined therapeutic strategies.

### 2.3. GO Functional Enrichment of PhA/WH Targets

**Figure 3:**
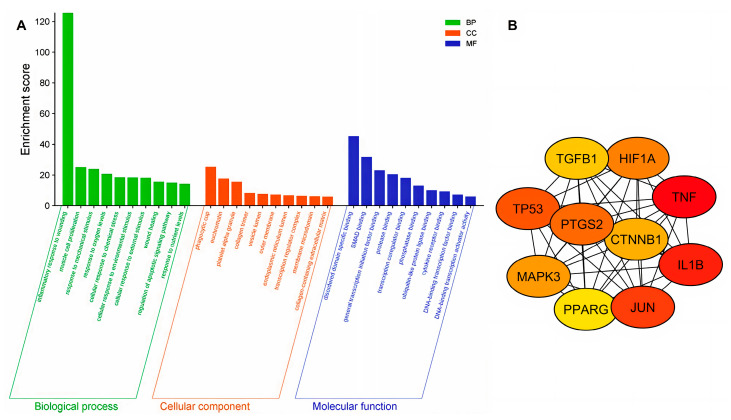
Functional and interaction analysis of differentially expressed genes. (**A**) Functional enrichment of differentially expressed genes according to the categories of biological processes (BPs), cellular components (CCs), and molecular functions (MFs). Enrichment scores reflect the relevance of the associated functions. (**B**) Protein–protein interaction (PPI) network of the identified key genes. Nodes represent the most relevant genes, with colors indicating their level of connectivity (red: highest connectivity; yellow: lowest connectivity).

To gain insight into the mechanisms associated with potential PhA-/WH-related targets, we performed GO functional enrichment analysis. The results, presented in Figure 3A, reveal that the inflammatory response to wounding, muscle cell proliferation, and response to mechanical stimuli were the main biological processes (BPs) enriched in relation to anti-PhA/WH targets.

Regarding cellular components (CCs), the most significant enrichment was observed in phagocytic cup, euchromatin, and platelet alpha granule. In the context of molecular functions (MFs), three categories were particularly prominent: binding associated with disordered domains, interactions involving SMAD proteins, and connections to general transcription initiation factors.

### 2.4. Construction of PPI Networks and Selection of Hub Proteins

A protein–protein interaction (PPI) network was constructed using the STRING database (https://cn.string-db.org/, accessed on 5 November 2024) by applying a confidence score threshold of 0.4. Network visualization was carried out using Cytoscape (version 3.10.3). Key modules were identified using the MCODE plugin, and the protein rankings within these modules were determined using CytoHubba.

To select hub proteins, the top ten proteins were identified using three CytoHubba algorithms: degree, closeness, and betweenness. The intersection of these results was used to obtain the final hub proteins: TNF, IL1B, JUN, PPARG, MAPK3, TP53, TGFB1, HIF1A, PTGS2, and CTNNB1 (Figure 3B). To complement these findings, we conducted a targeted literature and database search (PubMed, RCSB-PDB, DrugBank) to retrieve available docking or biophysical interaction data between AP and the ten hub targets [22,23,24,25,26,27,28]. For instance, TP53 exhibited a binding energy of −8.40 kcal/mol in PyRx, slightly stronger than the −7.8 kcal/mol reported in the literature [27]. Conversely, for MAPK3, PyRx predicted a weaker interaction (−7.70 kcal/mol) compared to the literature value of −8.9 kcal/mol using the same PDB ID (4QTB), which could reflect differences in grid box dimensions or scoring functions. Similarly, for PPAR-γ, PyRx showed a more favorable binding energy (−8.40 kcal/mol with 5Y2O) compared to −7.7 kcal/mol reported for a different structure (7AWC) in the literature [26]. The variation could be due to the different crystallographic conformations or co-crystallized ligands used in the docking studies. Interestingly, in the case of PTGS2, both the PyRx and literature results indicate strong binding (<−8.0 kcal/mol), supporting a consistent interaction profile. However, for TNF-α, the PyRx result (−6.70 kcal/mol with 7JRA) is weaker than the literature value (−7.52 kcal/mol with 5MU8, [25]), again suggesting potential methodological differences. For TGFB1, a greater discrepancy is observed, with PyRx estimating a more negative binding energy (−6.20 kcal/mol) compared to the literature’s −4.93 kcal/mol. This could reflect differences in ligand conformers, binding site definitions, or even protein flexibility considerations. Finally, docking against CTNNB1 showed identical binding energies in both approaches (−6.30 kcal/mol using 1JDH), reinforcing the consistency of results when docking parameters and structural inputs are matched. Compiled results are presented in Supplementary Figure S2 and Supplementary Table S1.

### 2.5. Effect of Andrographolide on PhA-/WH-Related Targets

**Figure 4:**
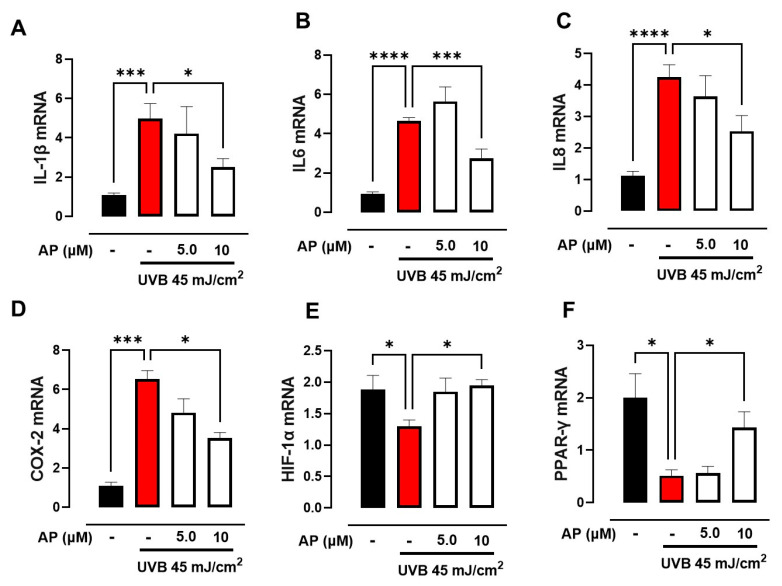
Effects of AP on the expression of genes associated with inflammation and metabolism in UVB-irradiated HaCaT cells. Relative mRNA expression levels of (**A**) IL-1, (**B**) IL-6, (**C**) IL-8, (**D**) COX-2, (**E**) HIF-1α, and (**F**) PPAR-γ in UVB-irradiated HaCaT cells treated with 5 and 10 µM AP for 6 h. Each bar represents mean ± SEM (*n* = 5). * *p* < 0.05; *** *p* < 0.001 and **** *p* < 0.0001 compared to the control UVB group.

Following the computational analysis of potential targets associated with photoaging and wound healing, we evaluated the effect of AP on HaCaT keratinocytes exposed to UVB radiation. In these experiments, we analyzed the expression of the pro-inflammatory cytokines IL-1β, IL-6, and IL-8. As expected, UVB irradiation significantly increased the levels of all three cytokines tested, whereas AP-treated cells showed markedly decreased expression of *IL-1β*, *IL-6*, and *IL-8* at a concentration of 10 µM (Figure 4A–C). Furthermore, ELISA results revealed a similar pattern in IL-6 and IL-8 secretion (Supplementary Figure S3A,B).

Moreover, our analysis of *COX-2* expression revealed a similar pattern, indicating that AP mitigated the increase induced by UVB irradiation (Figure 4D).

**Figure 5:**
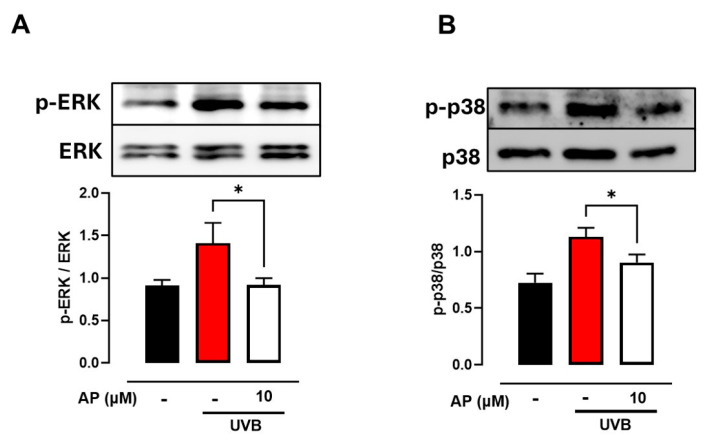
Effect of AP on UVB-induced ERK1/2 and P38 phosphorylation in HaCaT cells. HaCaT cells were irradiated with UVB and treated with 10 µM of AP. (**A**) Representative *p*-ERK1/2 immunoblot is shown, and densitometry values were quantified using the ImageJ software and normalized to ERK1/2. (**B**) Representative *p*-P38 immunoblot is shown, and densitometry values were quantified using Image Studio Lite v5.2 software and normalized to P38. Each bar represents mean ± SEM of at least 3 experiments. * *p* < 0.05 compared to the control UVB group.

Our study focused on analyzing the expression of two metabolism-related factors, *HIF-1α* and *PPAR-γ*. These results indicate that exposure to UVB radiation led to a substantial decrease in the expression levels of both HIF-1α and PPAR-γ in HaCaT keratinocytes. Nevertheless, AP administration successfully counteracted this reduction (Figure 4E,F). These findings suggest that AP may partially counteract inflammatory and metabolic disruptions induced by UVB exposure, highlighting its potential as a candidate for further investigation of photoaging-related wound repair. Hence, we investigated the effects of AP on the activation of the MAPK pathway components ERK1/2 and p38. Our results showed that UVB radiation induced MAPK activation, as evidenced by the increase in ERK1/2 and p38 phosphorylation in HaCaT keratinocytes compared to non-irradiated control cells (Figure 5A,B). However, treatment with 10 µM AP significantly inhibited phosphorylation of ERK1/2 and p38.

These findings suggested that AP modulated UVB-induced inflammatory and metabolic responses by inhibiting ERK1/2 and p38 activation over the time course analyzed in vitro.

### 2.6. Andrographolide Reverses UVB-Induced Metabolic Reprogramming

**Figure 6:**
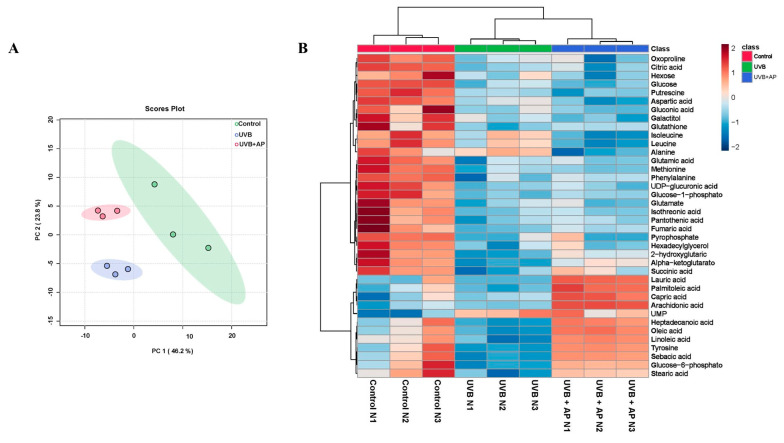
Metabolomic analysis of UVB-irradiated HaCaT cells with or without AP. (**A**) The principal component plot (PCA) showing the separation between the experimental groups: control, UVB, and UVB + AP. Each point represents a biological sample, whereas ellipses indicate variability within each group (95% confidence interval). This clear separation reflects the significant differences in the metabolic profiles between the conditions. (**B**) A heatmap of differentially expressed metabolites hierarchically grouped according to their relative abundance in the control, UVB, and UVB + AP groups. Colors represent the relative intensities of each metabolite (red: high; blue: low).

Skin wound healing involves metabolic reprogramming of nutrients such as glucose, lipids, and amino acids, which play vital roles in the proliferation, differentiation, and migration of multiple cell types [3,4]. It was recently shown that UVB radiation produces metabolic alterations in keratinocytes, characterized by the downregulation of glycolysis, tricarboxylic acid cycle, and fatty acid β-oxidation [3,4]. We investigated whether metabolic reprogramming could be reversed by AP treatment following UVB exposure. To achieve this, we studied the effect of 6 h after UVB irradiation at 45 mJ/cm^2^ on metabolite changes and AP treatment. We used multivariate analysis to define the metabolomic changes caused by UVB and AP treatment. Using Principal Component Analysis (PCA), we observed a variance value (R^2^X) of 0.73 and a predictive ability (Q2) of 0.81, indicating the excellence of the PCA model. From the PCA score plots, the metabolite profiles were clearly separated among the three groups (control, UVB, UVB + AP). Among the three groups, Principal Component (PC) 1 exhibited the maximum variability (46.2%), revealing substantial differences in metabolite profiles among the control, UVB-irradiated, and AP-treated groups (Figure 6A).

To evaluate the metabolic changes induced by exposure to UVB radiation and subsequent treatment with AP, we constructed a heatmap considering 38 metabolites in the control, UVB and UVB + AP groups (Figure 6B). We observed that UVB radiation increased the levels of amino acids such as alanine, aspartic acid, leucine, and isoleucine. In addition, a reduction in the critical fatty acids for skin maintenance, such as sebacic acid, palmitoleic acid, oleic acid, and lauric acid, were observed.

**Figure 7:**
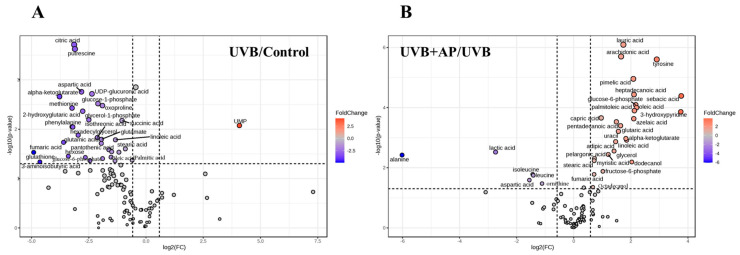
Volcano plots showing differentially expressed metabolites in UVB-irradiated HaCaT cells treated with or without AP. (**A**) Comparison of metabolites between the UVB and control groups. Significantly altered metabolites are highlighted by their position outside the cut-off lines (log2(FC) > 1 or <−1 and −log10 (*p*-value) > 1.3). (**B**) Comparison of metabolites between UVB + AP and UVB groups. Differentially expressed metabolites, such as fatty acids (palmitic, linoleic, and arachidonic acids) and amino acids (tyrosine and isoleucine), showed significant changes after AP treatment. The colors of the points reflect the fold change (FC), where red indicates upregulation, and blue indicates downregulation. These results highlight the key metabolites involved in UVB-induced metabolic modulation and their regulation by AP.

Volcano plots depict the patterns of differential expression among metabolites in both the control groups compared to the UVB-irradiated group and the UVB-irradiated group compared to the irradiated and AP-treated groups (Figure 7A,B). Each data point represented a distinct metabolite. The points on the left side of the plot indicate downregulated metabolites, whereas those on the right side indicate upregulated metabolites.

**Figure 8:**
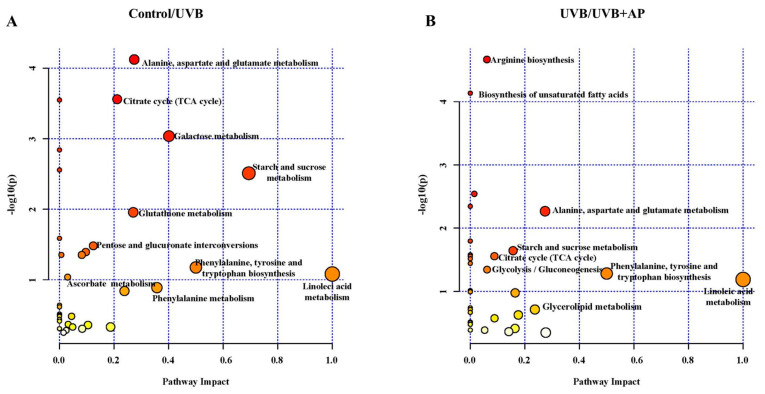
Metabolic pathway analysis of UVB-irradiated HaCaT cells treated with or without AP. (**A**) The impact of metabolic pathways differentially altered between the UVB and control groups. The main affected pathways included alanine, aspartate, and glutamate metabolism, Krebs cycle (TCA), and galactose metabolism, indicating alterations in energy and amino acid metabolism. (**B**) The impact of differentially altered metabolic pathways in the UVB + AP and UVB groups. The main modulated pathways include unsaturated fatty acid metabolism; alanine, aspartate, and glutamate metabolism; and lipid metabolism, suggesting PA-mediated metabolic regulation in response to UVB damage. Dots represent metabolic pathways, and their size and color reflect the magnitude of the impact (X-axis: pathway impact; Y-axis: statistical significance −log10(p)). The most important routes are marked with larger sizes and more intense colors.

Intermediate metabolites of the TCA cycle and glycolysis, such as glucose-6-phosphate, alpha-ketoglutarate, and fumaric acid, decreased in cells exposed to UVB radiation, whereas lactic acid levels increased in irradiated cells (Figure 7A,B). However, AP treatment increased the levels of both fatty acids and intermediate metabolites in the TCA cycle (Figure 7B). Pathway analysis revealed that alanine, aspartate, and glutamate metabolism, the TCA cycle, starch and sucrose metabolism, and glutathione (GSH) metabolism were affected by UVB (Figure 8A). Pathway analysis revealed that AP modulates arginine biosynthesis; alanine, aspartate, and glutamate metabolism; starch and sucrose metabolism; biosynthesis of unsaturated fatty acids; TCA cycle; and glycolysis/gluconeogenesis (Figure 8B).

## 3. Discussion

In this study, we assessed the effects of AP on wound healing in HaCaT cells exposed to UVB radiation. We demonstrated that AP improved the wound closure. A previous study showed that ASB, a water-soluble analog, attenuated UVB-induced photodamage and reduced the NF-kB pathway in HaCaT cells [20]. The effect of ASB has also been associated with the reduction in reactive oxygen species (ROS) induced by UVB and an increase in the NRF2 pathway [20]. Despite significant advances in research on the therapeutic benefits of AP in wound healing and photoaging, the molecular mechanisms underlying its effects on tissue repair under these conditions are not fully understood.

In this study, we used network pharmacology analysis to investigate the potential of AP to promote wound healing under photoaging conditions. This approach integrates small molecule analysis, molecular target identification, biological pathway enrichment, and protein–protein interaction studies. By employing this comprehensive methodology, we aimed to uncover the underlying mechanisms of action, provide a solid foundation for optimizing therapeutic applications, and develop more effective combinatorial treatment strategies.

Network pharmacology has emerged as a crucial tool for identifying therapeutic agents capable of targeting multiple interconnected factors and pathways, particularly in complex biological processes, such as wound healing, which are affected by oxidative stress and inflammation associated with photoaging. Its predictive capabilities facilitate the elucidation of the mechanisms of action and identification of potential molecular interactions, as evidenced by recent studies [15,29,30]. Our study initially analyzed 32 potential targets and identified 10 key targets linked to the anti-photoaging and wound-healing effects of AP: TNF, IL1B, JUN, TP53, MAPK3, PTGS2, HIF1A, TGFB1, CTNNB1, and PPARG. Gene Ontology (GO) analysis highlighted the inflammatory response to wounding as the most significant biological process involved. These findings emphasize the critical role of inflammatory pathways in tissue repair, particularly in the context of photoaging, and highlight the potential of targeting these pathways to enhance the therapeutic efficacy of AP.

UV radiation is widely recognized as the primary contributor to skin aging, leading to significant alterations in skin immune cells. These changes directly affect immune responses, amplify inflammation, and exacerbate skin tissue damage [31,32,33,34]. In our network pharmacology analysis, IL-1β and COX-2 were identified as key proteins mediating the therapeutic effects of AP in PhA/WH. It has been proven that sustained activation of the IL-1β pathway in diabetic wounds impairs the wound healing process by perpetuating inflammation. Inhibition of this pathway facilitates a shift from pro-inflammatory macrophages to healing-associated macrophages, thereby enhancing the production of wound growth factors and promoting improved healing outcomes [35,36].

Similarly, the selective inhibition of COX-2 in pressure ulcers has been reported to accelerate wound healing by reducing iNOS levels, which are associated with sustained inflammatory responses. These findings underscore the critical importance of reducing and regulating the expression of these pro-inflammatory molecules to minimize skin damage caused by ultraviolet radiation [36].

However, further research is required to elucidate the role of these inflammatory pathways in skin wounds associated with photoaging. This knowledge is essential to better understand the molecular mechanisms underlying the therapeutic potential of AP under such conditions and optimize its clinical applications.

Additionally, we observed that AP reduced the UVB-induced phosphorylation of ERK1/2 and p38 in HaCaT cells. Elevated ROS levels following UV exposure are well-known contributors to photoaging of the skin. ROS-mediated signaling pathways, including ERK and p38, can influence enzymatic expression and cytokine production, thereby exacerbating inflammatory and degenerative processes in the skin. UVB radiation can activate the MAPK pathway, leading to increased COX-2 expression via activation of AP-1 [37,38,39]. Moreover, the inhibition of p38 MAPK and AP-1 has been shown to reduce COX-2 expression, suggesting that pharmacological targeting of this pathway could be a viable strategy for treating UVB-induced inflammatory responses [22,38].

Several molecular targets have been proposed for AP, including the MAPK, PI3K/Akt, AP-1, NF-κB, PPAR-γ, and HIF-1 pathways [5,40,41,42]. In our network pharmacology analysis, PPAR-γ and HIF-1α were identified as the ten most relevant protein targets of AP in the treatment of PhA/WH. These findings suggested that these pathways play critical roles in mediating the therapeutic effects of AP. Specifically, they may serve as points of interaction between metabolic adaptation and photodamage in keratinocytes, highlighting the potential crosstalk between cellular energy regulation and the response to UV-induced stress. Further research is required to elucidate the precise mechanisms that link these pathways to the therapeutic effects of AP.

UVB irradiation induces biphasic modulation of HIF-1α, characterized by an early decrease followed by delayed upregulation [10,11,43]. This early reduction may impair cellular adaptive responses to oxidative stress, whereas a sustained increase may represent a compensatory mechanism. Similarly, PPARγ expression can also be transiently reduced following UVB exposure, contributing to increased inflammation, redox imbalance, and metabolic disruption [44]. Exposure to UVB radiation results in the production of reactive oxygen species, causing oxidative stress that can destabilize the HIF-1α protein and inhibit PPAR-γ transcription. Additionally, UVB triggers pro-inflammatory pathways such as NF-κB and AP-1, which adversely affect PPARγ and disrupt the transcriptional function of HIF-1α [10]. In addition, UVB-induced DNA damage activates p53, a key stress response factor that may further repress the expression of both HIF-1α and PPARγ [11,45].

The simultaneous downregulation of HIF-1α and PPARγ during the early phases of UVB-induced stress could compromise essential cytoprotective and homeostatic functions, thereby facilitating chronic inflammation, oxidative damage, and metabolic dysfunction, which are key features of photoaging.

Importantly, our findings suggest that AP may help rapidly restore the expression of both HIF-1α and PPARγ following UVB exposure. This early reactivation may be critical for preventing the establishment of chronic inflammatory and oxidative conditions. Mechanistically, this effect may be mediated through the PI3K/AKT and AMPK pathways, both of which are known to regulate HIF-1α and PPARγ activities and may be modulated by AP [41,46,47]. Interestingly, reports describe the inhibition of HIF-1α by AP under hypoxic conditions, in contrast to our findings, where AP restored HIF-1α expression is reduced by UVB, suggesting that the regulation of HIF-1α by AP is context-dependent and may serve distinct roles under oxidative versus hypoxic stress [48]. Moreover, by reducing ROS levels, AP may help to stabilize redox-sensitive transcription factors, further supporting metabolic reprogramming and cellular protection.

Taken together, we propose that AP facilitates the timely recovery of HIF-1α and PPARγ activity after UVB-induced suppression, thereby attenuating the inflammatory and metabolic disturbances that contribute to long-term skin damage and photoaging.

Previously, we demonstrated that UVB radiation inhibited amino acid metabolism and reduced key carbon metabolism pathways, including glycolysis/gluconeogenesis, citric acid cycle, pentose phosphate pathway, and GSH metabolism [4].

We demonstrated that AP treatment reversed UVB-induced metabolomic changes, with prominent effects on fatty acid biosynthesis, TCA cycle, and glycolysis/gluconeogenesis. Increases in key intermediates, such as glucose-6-phosphate, fructose-6-phosphate, and TCA cycle metabolites, such as alpha-ketoglutarate and fumarate, suggest significant recovery of glycolysis and mitochondrial activity. Furthermore, increases in essential fatty acids, such as sebacic acid, linoleic acid, oleic acid, and palmitoleic acid, indicate the restoration of lipid biosynthesis required for proper skin function. These fatty acids play a key role in the management of skin conditions such as atopic dermatitis, psoriasis, and rosacea [49,50,51].

Significant decreases in triglycerides (TG) and total free fatty acids (FFAs) in the epidermis were observed after acute and chronic exposure of human skin to UVB radiation. These changes in skin-free fatty acids after UV irradiation have been associated with fatty acid synthesis [52]. Ultraviolet irradiation can decrease the expression of lipogenic enzymes, such as acetyl-CoA carboxylase (ACC), fatty acid synthase (FAS), and stearoyl-CoA desaturase (SCD), as well as transcription factors, such as PPAR-γ and sterol regulatory element binding proteins (SREBP-2), leading to a reduction in FFA and TG synthesis in photodamaged skin [52]. Network pharmacology analyses and in vitro experiments revealed that AP effectively restored the reduced expression of PPARγ induced by UVB radiation in keratinocytes. AP exerts anti-inflammatory effects, partly through the activation and upregulation of PPARγ expression. Notably, PPARγ is also involved in lipid metabolism by regulating genes associated with the release, transport, and storage of fatty acids [52,53]. Recent studies have highlighted the significant role of PPARs in skin function. Specifically, PPARγ promotes de novo synthesis of triglycerides by inducing SREBP and the expression of its target genes [52,53]. These findings agree with previous studies showing that anti-inflammatory effects and reduction in UVB-induced lipid peroxidation are achieved by increasing the reduced levels of PPAR-γ and its activation [54]. These findings underscore the significance of PPAR-γ as a crucial factor in defense mechanisms against UVB radiation-induced damage.

Fatty acids play essential roles in maintaining the structural integrity and barrier function of the skin as well as in regulating inflammatory responses. In addition, oleic acid, a monounsaturated fatty acid, has been shown to accelerate wound healing, accompanied by a decrease in nitric oxide production at the wound site [55,56]. Furthermore, treatment with oleic acid enhances the levels of IL-10 in wounds, which correlates with the downregulation of COX-2. IL-10 plays a critical role in controlling inflammation by counteracting the pro-inflammatory effects of IL-17. This interplay helps regulate the inflammatory response in oleic acid-treated wounds, thereby promoting faster healing [55,56]. AP can also induce the production of IL-10 [40,41,42], and reduce IL-17, thereby attenuating inflammation in different animal models. In mice exposed to UVB irradiation at a dose of 180 mJ/cm^2^ for ten consecutive days, AP (3.6 mg/kg body weight) applied topically 1 h before each UVB exposure reduced inflammatory proteins like CD34, iNOS, NF-κB, COX-2, IL-6, and IL-10 in the skin [57,58].

Medium-chain triglycerides (MCTs) are synthesized in the human body from medium-chain fatty acids (MCFAs), such as lauric acid, myristic acid, and glycerin [59,60]. Both fatty acids decreased in UVB-irradiated cells; however, AP treatment restored the levels of both fatty acids. Studies have shown that topical applications of MCT and linoleic acid (LA) can improve wound healing in mice, involving processes such as inflammation, cell proliferation, contraction, and tissue remodeling [59,60]. These findings suggest that MCFAs can accelerate human healing. In line with this, clinical reports have suggested that essential fatty acid deficiency (EFAD) may cause delayed healing [59]. The positive effect of fatty acids on wound healing is related to their antioxidant and anti-inflammatory effects in the later inflammatory phase of tissue repair, favoring the initiation of the proliferative phase [60].

Omega-6 polyunsaturated fatty acids, linoleic acid among them, have been found to exhibit antioxidant effects in hepatocytes via the Kelch-like ECH-associated protein 1 (KEAP1)-NRF2 system, according to recent studies. However, the specific mechanisms and signaling pathways involved in this process are still not completely understood [61,62]. The p38 MAPK/NRF2/HO-1 signaling pathway has emerged as a significant protective mechanism against oxidative stress. Research on plant-derived natural products has demonstrated a decrease in the inflammatory cytokines IL-1β and TNF-α through this pathway as well as the activation of superoxide dismutase (SOD) and GSH [61]. HaCaT cells irradiated with UVB showed decreased GSH levels, which was reversed by AP treatment. Accordingly, AP activates the transcription factor NRF2 [16] and expression of target antioxidant genes, supporting its antioxidant properties [5]. Furthermore, AP inhibits activation of the p38 MAPK pathway by inhibiting downstream signaling pathways involved in the inflammatory response that are transcriptionally regulated by AP-1 [5].

Inhibition of inflammatory responses in tissues plays a crucial role in limiting apoptosis and supporting cellular recovery [63]. AP has demonstrated notable anti-inflammatory activity in various cell types and is often associated with enhanced cell survival and reduced apoptotic signaling. Its water-soluble derivative, andrographolide sodium bisulfate, has also been shown to attenuate UVB-induced apoptosis in HaCaT keratinocytes, reinforcing its protective potential under conditions of oxidative stress [15,16,57,58]. In our study, AP treatment markedly reduced UVB-induced inflammatory responses. This anti-inflammatory effect, combined with the known antioxidant properties of AP, may contribute to an intracellular environment conducive to DNA repair and cell preservation. A key mechanism potentially involved in this protective response is regulation of the p38 MAPK pathway. UVB irradiation activates p38, a kinase closely linked to pro-apoptotic signaling, particularly in cells with significant DNA damage. Persistent activation of p38 not only promotes apoptosis but also exacerbates oxidative stress [6,7]. In contrast, its inhibition has been associated with decreased ROS production and reduced oxidative DNA damage, including the formation of 8-oxo-dG (8-oxo-2′-deoxyguanosine), a hallmark of nucleotide oxidation. Moreover, attenuating p38 activation enhances the function of DNA repair proteins, promotes the resolution of genomic lesions, and contributes to cellular homeostasis [6,7].

Taken together, these findings suggest that AP may exert cytoprotective effects in UVB-exposed keratinocytes by modulating inflammatory signaling and promoting intracellular conditions favorable for oxidative damage control and DNA repair. Attenuation of p38 MAPK activation appears to play a role in limiting stress-induced responses that compromise cell viability and tissue integrity.

In addition to its anti-inflammatory and antioxidant effects, AP has been shown to modulate key metabolic pathways, particularly those involved in lipid metabolism. This activity may contribute to the restoration of keratinocyte migration disrupted by UVB exposure, an essential step in wound healing. These findings suggested that AP may influence cellular responses to UVB damage by targeting metabolic regulators. However, further studies are required to validate these pathways before considering their therapeutic applications. Metabolic reprogramming of glucose, lipids, and amino acids plays a central role in skin repair and regeneration [64], and several signaling molecules involved in these pathways are emerging as potential targets for enhancing wound healing [34]. Our data support the idea that bioactive compounds such as AP can selectively modulate these processes, offering insight into new molecular targets relevant to cutaneous damage and repair.

**Figure 9:**
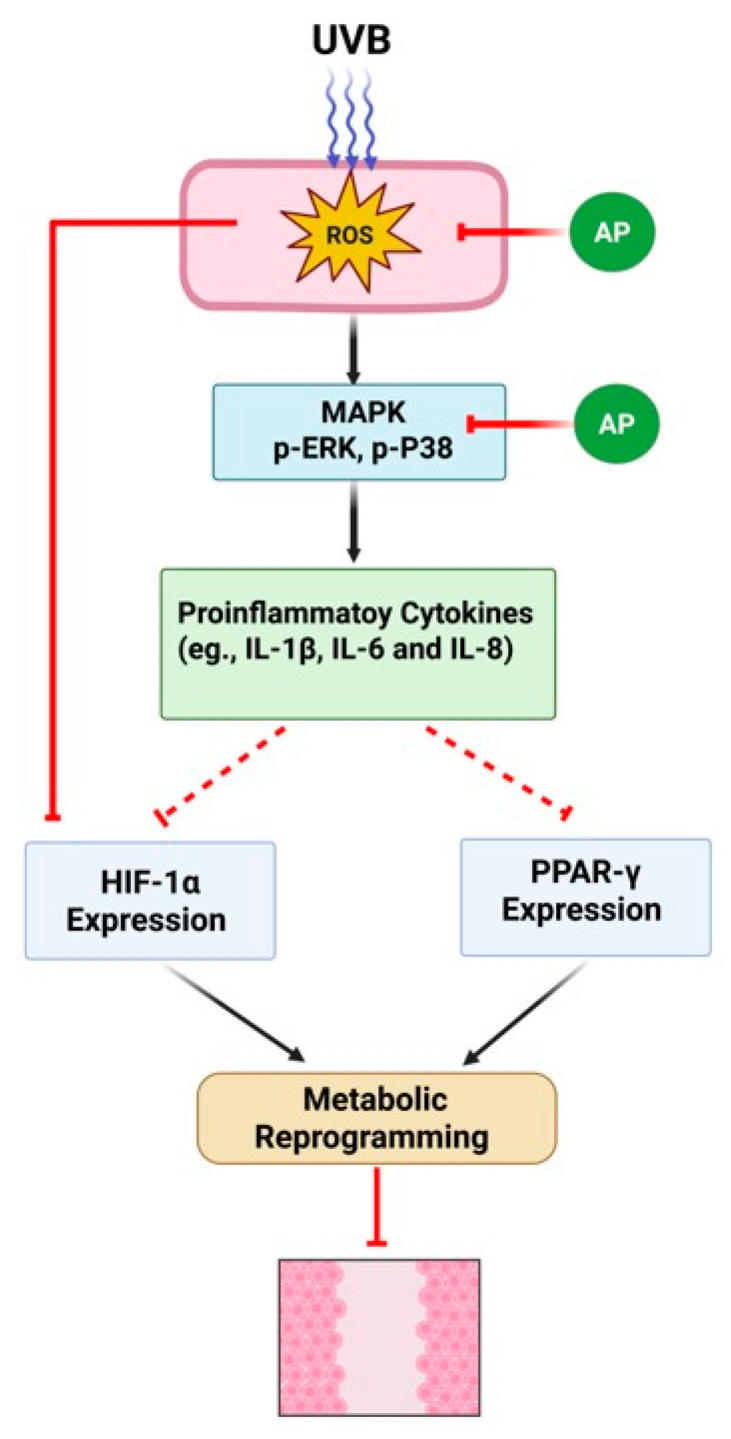
Schematic representation of the potential molecular mechanisms by which AP modulates UVB radiation-induced responses in keratinocytes. UVB radiation exposure induces oxidative stress (ROS), activates the MAPK signaling pathway (ERK1/2 and p38 phosphorylation), and increases the expression of pro-inflammatory genes (*IL-1β*, *IL-6*, *IL-8*, and *COX-2*). Furthermore, UVB reduces the expression of the transcription factors *HIF-1α* and *PPARγ,* which could contribute to the disruption of key metabolic pathways, such as glycolysis, amino acid metabolism, and fatty acid metabolism. AP treatment attenuated UVB-induced ERK1/2 and p38 phosphorylation, decreased the expression of inflammatory genes, and restored *HIF-1α* and *PPARγ* levels, thereby promoting the restoration of metabolic balance. UVB also reduced cell migration in the scratch assay, an effect that was reversed by AP, possibly through its anti-inflammatory action and the metabolic reprogramming that it induced. The dotted lines indicate potential effects or modulations that were not directly confirmed in this study. The solid lines indicate the effects experimentally confirmed in this study.

This study highlights the modulatory effects of AP on the key inflammatory and metabolic pathways involved in skin repair under photoaging conditions (Figure 9). Using an in vitro model of keratinocytes, we identified potential pharmacological targets and mechanisms through which AP may promote tissue repair, including the regulation of pro-inflammatory genes (*IL-1β* and *COX-2*) and transcription factors central to carbohydrate and lipid metabolism (*HIF-1α* and *PPARγ*). These findings contribute to our understanding of how bioactive compounds influence metabolic reprogramming and inflammatory responses during wound healing.

However, this study has some limitations such as the exclusive focus on a single cell type as well as the lack of co-culture experiments. Wound healing is a multifaceted process that requires the coordinated activity of multiple cell types and tissue components [34]. Moreover, functional assays to establish direct causal links between UVB-induced metabolic alterations and the restoration of inflammatory or reparative pathways by andrographolide were not performed. Although our integrative approach—combining untargeted metabolomics, gene/protein expression, and migration assays—provides strong correlative evidence, further studies using gene silencing, pharmacological inhibitors, or target overexpression will be essential to confirm the underlying mechanisms.

Therefore, although our results provide valuable mechanistic insights, further research using more complex models is essential to fully assess the therapeutic potential of AP and its relevance in vivo.

## 4. Materials and Methods

### 4.1. Cells Culture

HaCaT cells, a line of immortalized human keratinocytes [65], were generously provided by Dr. Miguel Concha of the Department of Pathology, Faculty of Medicine, Universidad Austral de Chile, Valdivia, Chile. HaCaT cells are a spontaneously immortalized human keratinocyte line that preserves the ability to differentiate and closely mimics epidermal keratinocyte responses under physiological and stress conditions. Owing to their reproducible responses to UVB irradiation—such as ROS generation, cytokine release (e.g., IL-6, TNF-α), and activation of key signaling pathways (NF-κB, MAPKs)—they are extensively used to study photoaging and inflammation in vitro [66,67].

The cells were maintained in Dulbecco’s modified Eagle’s medium (DMEM; HyClone, GE Healthcare Life Sciences, Logan, UT, USA) supplemented with 10% fetal bovine serum (FBS; Gibco, Grand Island, NY, USA) and antibiotics [100 U/mL penicillin and 100 µg/mL streptomycin (Invitrogen, Thermo Fisher Scientific, Waltham, MA, USA)] at 37 °C in a humidified incubator with 5% CO_2_. HaCaT cells were allowed to proliferate until they reached 90% confluence, at which point they were subcultured on appropriate plates for experimental assays. Prior to all the experiments, the cells underwent a 24 h incubation period in serum-free DMEM.

### 4.2. Cell Viability Assay

Cell viability was assessed using the Cell Counting Kit-8 (CCK-8; #96992, Sigma-Aldrich, St. Louis, MO, USA) according to the manufacturer’s instructions. HaCaT cells were seeded in 96-well plates and cultured until reaching confluency. Subsequently, cells were incubated in serum-free medium for 24 h. To evaluate the cytotoxicity of andrographolide, cells were exposed to increasing concentrations (5, 10, 20, 30, and 50 µM) and incubated for an additional 24 h. Following treatment, 10 µL of CCK-8 reagent was added to each well, and plates were incubated for 2 h at 37 °C in a humidified incubator with 5% CO_2_. Absorbance was measured at 450 nm using a microplate reader (Stat Fax^®^ 2100, Palm City, FL, USA), and cell viability was expressed as a percentage relative to untreated controls.

### 4.3. UVB Irradiation

For UVB irradiation, HaCaT cells were cultured in 12-well plates (1 × 10^5^ cells/well) for 24 h until they reached 80% confluence. Before irradiation, the cells were washed twice with phosphate-buffered saline (PBS), and a thin layer of solution was left to prevent the cell surfaces from drying. The cells were irradiated at 45 mJ/cm^2^ with a 3UV series UV hand lamp (UVP, LLC, Upland, CA, USA), which emitted 302 nm radiation. The irradiation doses were calculated using the following formula: dose (mJ/cm^2^) = exposure time (s) × intensity (mW/cm^2^) [32,68]. AP treatment was performed immediately after irradiation, and the cells were incubated for six hours under the same conditions as described above. AP was dissolved in dimethyl sulfoxide (DMSO), and the controls were treated with vehicle under the same conditions.

### 4.4. Scratch Wound Assay

HaCaT cells were plated in 12-well plates at a density of 2 × 10^5^ cells/mL per well and incubated at 37 °C until they reached full confluence. To halt cell division, mitomycin-C was introduced at 10 µg/mL, followed by a 2 h incubation. The cells were then washed and exposed to 45 mJ/cm^2^ UVB radiation. A sterile 20 µL pipette tip was used to create a uniform cell-free area across each confluent monolayer. After another wash, the detached cells in PBS were removed, and fresh medium containing either 5 µM or 10 µM AP (Sigma-Aldrich, St. Louis, MO, USA) was added to each well. The concentrations selected were based on prior studies using MTT assays, which demonstrated that AP at 5 µM and 10 µM had no detrimental effects on cell viability [4,5]. Cell migration into the wound area was observed at 0 and 24 h post-wounding using an inverted microscope with a digital camera attachment. Images were analyzed using ImageJ 1.35s software. Wound closure was calculated as the difference in the wound area between 0 and 24 h. For each sample, a minimum of five randomly selected scratched regions were assessed, and the mean values were determined.

### 4.5. Collection of Genes Associated with Photoaging (PhA) and Wound Healing (WH)

GeneCards (https://www.genecards.org/, accessed on 5 November 2024) and Online Mendelian Inheritance in Man (OMIM, https://www.omim.org, accessed on 5 November 2024) were used to build a comprehensive collection of genes associated with photoaging. Similarly, to identify genes associated with wound healing, a search was conducted in four public online databases: GeneCards (https://www.genecards.org/, accessed on 5 November 2024), Online Mendelian Inheritance in Man (OMIM, https://www.omim.org, accessed on 5 November 2024), DisGeNET (https://www.disgenet.org, accessed on 5 November 2024), and Comparative Toxicogenomics Database (CTD, https://ctdbase.org, accessed on 5 November 2024).

After collecting data from both searches, a rigorous deduplication process was applied to remove redundant records and ensure accurate selection of relevant molecules related to both photoaging (PhA) and wound healing (WH). Subsequently, an overlay of the genes from both groups was performed to identify a final set of common genes, named ‘Potential Targets of Photoaging vs. Wound Healing’ (PhA/WH). Visualization of the data overlay was carried out using the Venn online tool (https://bioinformatics.psb.ugent.be/webtools/Venn/, accessed on 5 November 2024), which allowed the identification and highlighting of key intersections between the analyzed gene sets.

### 4.6. Drug Target Prediction for PhA/WH vs. Andrographolide

The identification of AP drug targets was based on information reported by other authors and widely recognized prediction tools [15]. Briefly, the 3D molecular structure file and canonical smile representation of AP were imported into the following platforms: Swiss Target Prediction (https://www.expasy.org/resources/swisstargetprediction, accessed on 5 November 2024), SuperPred (https://prediction.charite.de/, accessed on 5 November 2024), PharmMapper (https://lilab-ecust.cn/pharmmapper/index.html, accessed on 5 November 2024), and Comparative Toxicogenomics Database (CTD) (https://ctdbase.org, accessed on 5 November 2024). These tools are web servers that specialize in the identification of potential drug targets. They use extensive and updated databases to predict potential drug interactions, making them a reliable and widely used resource in scientific research.

After identifying the potential targets of AP, the data were normalized using the UniProt database (https://www.uniprot.org/, accessed on 5 November 2024) to ensure consistency and accuracy in the annotations of the target proteins.

Overlap analysis was performed using the Venn online tool (https://bioinformatics.psb.ugent.be/webtools/Venn/, accessed on 5 November 2024) to identify the common targets of PhA-/WH-associated genes and AP pharmacological targets.

This approach allowed us to determine possible molecular intersections and shared therapeutic targets, providing information on the potential mechanisms of AP in processes related to photoaging and wound healing.

### 4.7. Gene Ontology Functional Enrichment and Visualization of PhA/WH Targets

Functional enrichment analysis of PhA/WH targets was performed using annotation and enrichment analysis tools in Gene Ontology (GO) databases. These tools allowed for the identification of relevant biological functions and metabolic pathways involved in the targets studied. For the analysis, the WebGestalt bioinformatics platform (https://www.webgestalt.org/, accessed on 5 November 2024) was used, which facilitates a comprehensive and automated approach to functional enrichment. The results of the analysis were organized using unsupervised hierarchical clustering, which allowed for clear and efficient categorization of the data obtained. The enrichment results were visualized using bar graphs, providing a clear and accessible representation of the most significant terms and pathways.

The GO database allowed the functional classification of genes and proteins into three main domains: Biological processes (BPs), which describe the biological activities involved in specific functions. Cellular components (CCs): identify the locations within the cell where genes or proteins perform their functions. Molecular functions (MFs): details of biochemical activities performed by gene products.

A *p* value < 0.05 was considered a statistical significance criterion for functional annotation in GO. This threshold ensured that the results reflected robust and biologically relevant associations. This approach allowed for the identification of key biological functions and metabolic pathways associated with PhA/WH targets, providing critical information for understanding the underlying molecular mechanisms and potential therapeutic strategies.

### 4.8. Construction of PPI Networks and Selection of Hub Proteins

A protein–protein interaction (PPI) network was constructed using the STRING database (https://cn.string-db.org/, accessed on 5 November 2024) with a confidence score threshold of 0.4, ensuring the inclusion of high-probability interactions. Network visualization was performed using Cytoscape (version 3.10.3), a widely used platform for biological network analysis.

The MCODE plugin (v2.0.3) was used to identify key modules within the network, which allowed the detection of densely connected clusters representing possible functional complexes or important pathways. Subsequently, proteins in the key modules with the highest scores were ranked using the cytoHubba plugin (v0.1), a tool integrated into Cytoscape designed to assess the importance of proteins in PPI networks.

The selection of hub proteins was performed by applying three different algorithms in cytoHubba: Degree: Measures the direct connectivity of a protein in the network. Closeness: Evaluates how central a protein is to all others in the network. Betweenness: Determines how often a protein acts as a bridge between other proteins in the network.

The top ten hub proteins were selected according to each algorithm, and the final hub proteins were identified by intersecting the lists generated by the three methods. This systematic approach highlighted key proteins with potentially critical roles in underlying biological processes.

### 4.9. Quantitative Real-Time RT-PCR (qRT-PCR)

Total RNA was isolated from HaCaT cells using the E.Z.N.A. Total RNA Kit I (R6834-01; Omega Bio-Tek, Norcross, GA, USA). Genomic DNA was eliminated by treating the RNA with a Turbo DNase-Free kit (AM1907, AmbionTM, Thermo Fischer Scientific, Waltham, MA, USA). For cDNA production, 200 ng of RNA underwent reverse transcription using M-MLV reverse transcriptase (M5313, Promega, Madison, WI, USA) as per the manufacturer’s instructions. RT-qPCR was conducted using Takyon Rox SYBR MasterMix (UF-RSMT-B0701, Eurogentec, Seraing, Belgium) and the following primer sets: IL-1β F-5′TCCTGCGTGTTGAAAGATGATAA3′ and R-5′CAAATCGCTTTTCCATCTTCTTC3′; IL-6 F-5′TACCCCCAGGAGAAGATTCC3′ and R-5′GCCATCTTTGGAAGGTTCAG3′; IL-8 F-5′ACTGCTCAACACCGGAATTT3′ and R-5′TCCAAAATCCCTTGAAGTGGG3′; COX-2 F-5′ACTGCTCAACACCGGAATTT3′ and R-5′TCCAAAATCCCTTGAAGTGGG3′; HIF-1α F-5′GGCGCGAACGACAAGAAA3′ and R-5′GATTCTTTACTTCGCCGAGATCTG3′; PPARG F-5′GGATCCCTCCTCGGAAATGG3′ and R-5′TGGCTTCTTTCAAATCTGGTGT3′; RPS9 F-5′ACGCTTGATGAGAAGGACCC3′ and R-5′ATCTGCCCTCATCCAGCAC-3′.

RT-qPCR was performed on a StepOnePlus real-time PCR system (Applied Biosystems, Thermo Fisher Scientific, Waltham, MA, USA) using the following cycling protocol: initial denaturation at 95 °C for 10 min, followed by 40 cycles of denaturation at 95 °C for 30 s, annealing at 60 °C for 30 s, and extension at 72 °C for 30 s. Changes in expression were determined using the 2^−(ΔΔCt)^ method as described by Livak and Schmittgen [69]. RPS9 served as the housekeeping gene for normalization.

### 4.10. IL-6 and IL-8 Quantification via Enzyme-Linked Immunoassay (ELISA)

The supernatants collected from the qPCR assay were centrifuged at 600× *g* for 6 min and used to quantify IL-6 and IL-8 levels using ELISA kits (IL-6 #DY206-05; IL-8 #DY208-05, DuoSet™ ELISA, NE, Minneapolis, MN, USA), according to the manufacturer’s instructions. Capture antibodies were added to 96-well plates and incubated overnight at room temperature. The wells were then washed three times with PBS, and samples were added, followed by a 1 h incubation with blocking solution at room temperature. After additional washes, detection antibodies, streptavidin-HRP, and the substrate were sequentially added, with washing steps between each addition. The reaction was stopped with 0.16 M H_2_SO_3_, and absorbance was measured at 450 nm using a plate reader (Stat Fax^®^ 2100, Palm City, FL, USA). Data were analyzed using the GraphPad Prism software (version 8.0).

### 4.11. Western Blot Analysis

The cells were washed twice with PBS before lysis in radioimmunoprecipitation assay (RIPA) buffer containing phosphatase and protease inhibitors (A32963, Thermo Scientific). To eliminate cellular debris, the lysate was centrifuged at 17,000× *g* and 4 °C for 20 min. Protein concentrations were quantified using the Bradford assay. Equivalent amounts of protein were mixed with Laemmli buffer, heated at 95 °C for 5 min, separated on 10% polyacrylamide gels, and transferred to nitrocellulose membranes. Membranes were blocked with 5% skim milk in TBS-T (20 mM Tris-HCl, pH 7.5; 137 mM NaCl; 0.1% Tween 20) for 2 h. Membranes were then incubated with primary antibodies (1:1000) overnight at 4 °C. The primary antibodies used were anti-p-p38 monoclonal (sc-166182; Santa Cruz Biotechnology, Dallas, TX, USA), anti-p38 monoclonal (sc-81621; Santa Cruz Biotechnology, Dallas, TX, USA), anti-p-ERK monoclonal (sc-7383; Santa Cruz Biotechnology, Dallas, TX, USA), and anti-ERK1/2 monoclonal (sc-514302; Santa Cruz Biotechnology, Dallas, TX, USA). Housekeeping proteins correspond to the non-phosphorylated proteins ERK and p38. The following day, membranes were treated with HRP-conjugated anti-mouse IgG antibody (115-035-003; Jackson ImmunoResearch, West Grove, PA, USA). An enhanced chemiluminescence system (UVP ChemiD-itTS2, Upland, CA, USA) was used to visualize specific bands. The band density was analyzed using the ImageJ 1.35s software.

### 4.12. Untargeted Metabolomics Analysis by GC-MS

Samples that underwent derivatization (2 µL) were introduced into an Agilent 7890 B GC system linked to a 5977A mass-selective detector system operating in the electron impact ionization (EI) mode (Agilent Technologies, Palo Alto, CA, USA). An Agilent 7693 series autosampler (Agilent Technologies, Palo Alto, CA, USA) was used to inject the samples onto a 30 m × 0.25 mm × 0.25 μm DB-5 column (Agilent Technologies, Palo Alto, CA, USA). The injection port was maintained at 250 °C, and the helium carrier gas flow was maintained at 1 mL/min. The initial oven temperature was set to 60 °C and subsequently increased at a rate of 10 °C /min until it reached 325 °C, with a total run time of 37.5 min. Following a 5.9 min solvent delay, complete spectra (50–600 *m*/*z*; 1.7 scans/s) were obtained at a digital scan rate of 20 Hz, with the MS ion source and quadrupole temperatures set to 250 °C and 150 °C, respectively. Metabolite identification was performed according to the method outlined by Fiehn [70].

The data processing workflow involved peak detection, deconvolution, and peak alignment using MSDIAL 2.83 (RIKEN Center for Sustainable Resource Science: Metabolome Informatics Research Team, Yokohama, Japan). Each trimethylsilylated metabolite was examined during deconvolution. The resulting deconvoluted peaks were then compared against the mass spectral libraries in NIST MSP format for identification. The ranking of the library match hits in relation to the experimental data was based on the combined retention index and mass spectral similarity across all the processed samples within a batch.

The retention index employed was Fiehn’s RI, which was derived from FAME. Metabolite identification involved matching the EI spectra with reference compounds from either NIST or Fiehn libraries. The analysis parameters included a 2000 retention index tolerance, 70% EI similarity threshold, 70% identification score threshold, 0.5 Da *m*/*z* tolerance, and a 0.5 min retention time margin.

### 4.13. Molecular Docking Protocol

Molecular docking was carried out to investigate the interaction between andrographolide and the ten hub targets. The crystal structures of selected human receptors were retrieved from the RCSB Protein Data Bank (https://www.rcsb.org/, accessed on 5 November 2024), and their resolutions ranged from 1.4 Å to 3.0 Å. The list included TP53 (PDB ID: 4HJE, 1.91 Å), MAPK3 (4QTB, 1.40 Å), JUN (5FV8, 1.99 Å), HIF-1α (3HQU, 2.30 Å), PPAR-γ (5Y20, 1.80 Å), PTGS2 (5KIR, 2.70 Å), TNF-α (7TJRA, 3.00 Å), TGFB1 (5VQP, 2.90 Å), IL1B (6Y8M, 1.90 Å), and β-catenin/CTNNB1 (1JDH, 1.90 Å). The ligand andrographolide (PubChem CID: 5318517) was obtained and converted to PDBQT format using Open Babel integrated within PyRx software (version 0.8). Protein and ligand preparation included the removal of water molecules, addition of polar hydrogens, and assignment of Gasteiger charges.

The ligand–protein interaction was visualized using PyMOL (version 3.1.6.1), allowing for detailed analysis of the binding conformation and identification of residues involved in ligand interaction.

### 4.14. Statistical Analysis

Metabolite normalization for metabolomic analysis was performed using ribitol as the internal standard. Data were subjected to logarithmic transformation and autoscaling before statistical evaluation. MetaboAnalyst v 6.0 (Xia Lab, McGill University, Montreal, QC, Canada; http://www.metaboanalyst.ca, accessed on 5 November 2024) facilitated multivariate statistical examination. Euclidean distance measurement and Ward’s clustering algorithm were employed to construct a heatmap.

Pathway analysis incorporated only metabolites, demonstrating significant differences in topology. The experimental procedures were replicated at least three times using cells from distinct clones. Volcano plots showed that the metabolites exhibited a fold-change of ≥1.5. Additional results are presented as bar graphs, with error bars indicating ± S.E.M. Statistical analysis included one-way analysis of variance (ANOVA) followed by Fisher’s least significant difference (LSD) multiple comparison test. In cases where normality or variance homogeneity assumptions were violated, as determined by the Shapiro–Wilk or Brown–Forsythe tests, Kruskal–Wallis ANOVA and Dunn’s multiple comparison tests were implemented.

GraphPad Prism v7.0 (GraphPad Software, La Jolla, CA, USA) was used for all statistical assessments, with *p* < 0.05 established as the significance threshold.

## Data Availability

The original contributions presented in this study are included in the article and Supplementary Materials. Further inquiries can be directed to the corresponding author.

## Supplementary Materials

The following supporting information can be downloaded at https://www.mdpi.com/article/10.3390/ijms26136508/s1.
