# Supplementary material for: Andrographolide Mitigates Inflammation and Reverses UVB-Induced Metabolic Reprogramming in HaCaT Cells
Source: Int J Mol Sci. 2025 Jul 6;26(13):6508. doi: 10.3390/ijms26136508 (PMC12249694; doi:10.3390/ijms26136508)
Supplement: Supplementary file 1 [file ijms-26-06508-s001.zip › Supplementary material.pdf]

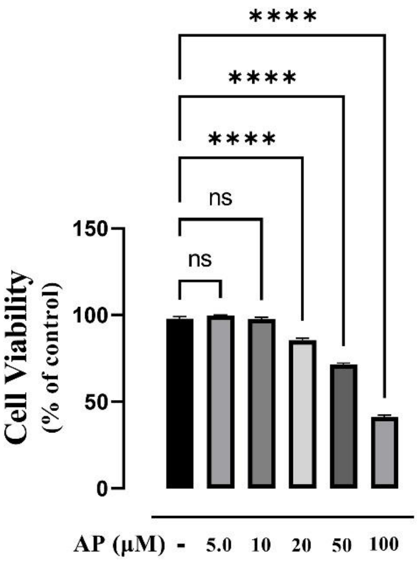

Supplementary Figure 1: Effect of andrographolide (AP) on cell viability. Cells were treated with AP for 24 h, and viability was assessed relative to untreated controls. Data are presented as mean ± SEM ($n = 5$). Statistical significance was determined using one-way ANOVA. ****$p < 0.0001$; ns: not significant.

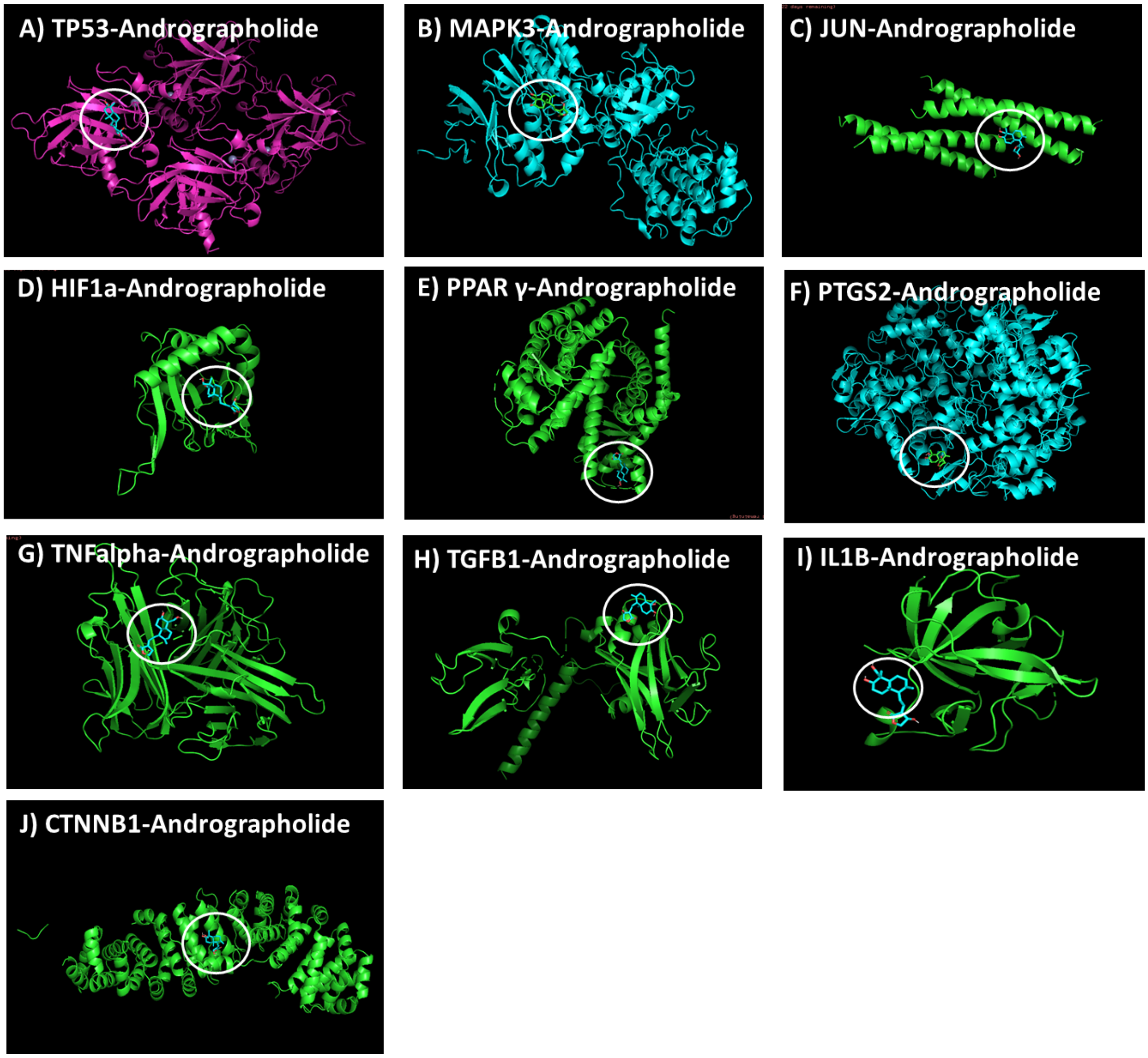

Supplementary Figure 2: Structural visualization of andrographolide binding to selected hub target proteins following molecular docking. Three-dimensional structural models of andrographolide docked to key hub target proteins, generated using PyRx and visualized in PyMOL. The protein structures—TP53, MAPK3, JUN, HIF-1α, PPAR-γ, PTGS2, TNF-α, TGFB1, IL1B, and CTNNB1—are represented in surface or ribbon format, while andrographolide is shown within the predicted binding pocket, enclosed within a white circle for emphasis. Protein chains are color-coded for clarity, and each complex is oriented to optimally expose the ligand-protein interface. The models facilitate qualitative evaluation of ligand positioning and provide visual confirmation of the molecular interactions predicted by docking analysis.

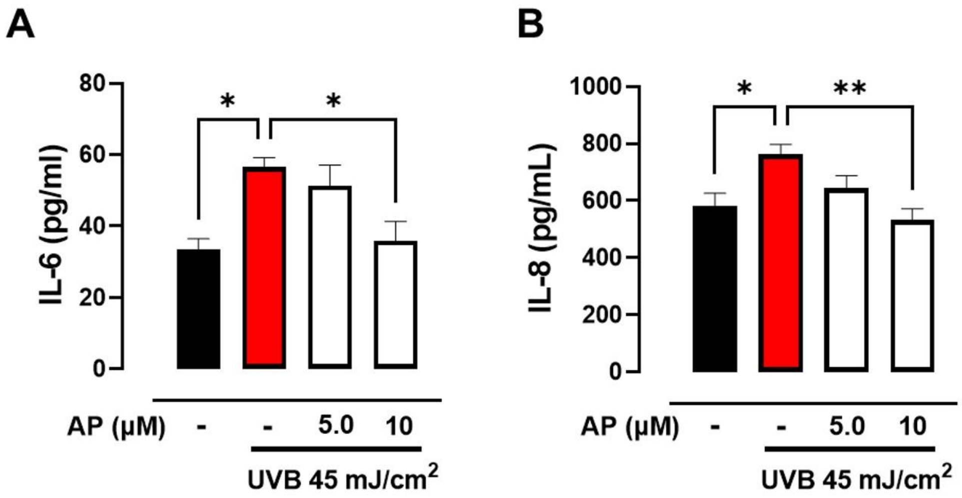

Supplementary Figure 3. **Effects of AP on IL-6 (A) and IL-8 (B) gene expression in UVB-irradiated HaCaT cells**. After UVB irradiation, cells were incubated for 6 h with andrographolide (AP). A) IL-6 and (B) IL-8 concentrations were assessed by ELISA. Data are presented as the mean ± SEM of at least three experiments. *$p < 0.05$ and **$p < 0.01$ compared to the UVB group.

Table 1 supplementary. Docking parameters and literature comparison for andrographolide and selected protein targets.

Target	PDB ID	Binding energy kcal/Mol (PyRx)	PDB ID of literature	Binding energy kcal/Mol of literature	References
**TP53**	4HJE	-8.40	3Q05	− 7.8	[27]
**MAPK3**	4QTB	-7.70	4QTB	− 8.9	[27]
**JUN**	5FV8	-5.8	5FV8	− 6	[27]
**HIF-1α**	3hqu	-6.30	3hqu	-6.5	[23]
**PPAR-γ**	5Y2O	-8.40	7AWC	-7.7	[26]
**PTGS2**	5KIR	-8.00	5F19	< - 8.0	[22]
**TNF-α**	7JRA	-6.70	5MU8	-7.52	[25]
**TGFB1**	5VQP	-6.20	5VQP	-4.93	[28]
**IL1B**	6Y8M	-5.10	6Y8M	−6.79	[24]
**CTNNB1**	1JDH	-6.30	1JDH	NR	

NR: not reported
